# Case Report: Detection of a Novel Germline *PALB2* Deletion in a Young Woman With Hereditary Breast Cancer: When the Patient's Phenotype History Doesn't Lie

**DOI:** 10.3389/fonc.2021.602523

**Published:** 2021-02-24

**Authors:** Carmine De Angelis, Carmela Nardelli, Paola Concolino, Martina Pagliuca, Mario Setaro, Elisa De Paolis, Pietro De Placido, Valeria Forestieri, Giovanni Luca Scaglione, Annalisa Ranieri, Barbara Lombardo, Lucio Pastore, Sabino De Placido, Ettore Capoluongo

**Affiliations:** ^1^Department of Clinical Medicine and Surgery, University of Naples Federico II, Naples, Italy; ^2^Department of Molecular Medicine and Medical Biotechnologies, University of Naples Federico II, Naples, Italy; ^3^Molecular and Genomic Diagnostics Unit, Fondazione Policlinico Universitario A. Gemelli Istituto di Ricovero e Cura a Carattere Scientifico, Rome, Italy; ^4^CEINGE-Biotecnologie Avanzate, Naples, Italy

**Keywords:** hereditary breast cancer, PALB2, breast-cancer risk, deletion, surveillance

## Abstract

The partner and localizer of *BRCA2* (*PALB2*) is a major *BRCA2* binding partner that participates in homologous recombination repair in response to DNA double-strand breaks. Germline alterations of the *PALB2* gene have recently been associated with a high risk of developing breast cancer. We investigated a 37-year-old Caucasian woman with breast cancer and family history of breast cancer using targeted next generation sequencing. A novel heterozygous deletion involving exons 5 and 6 was found in the *PALB2* gene, and resulted in the production of a truncated PALB2 protein. These findings expand the mutational spectra of *PALB2*-associated breast cancer, and may improve the mutation-based screening and genetic diagnosis of breast cancer.

## Background

Breast cancer is the most frequently diagnosed cancer and the leading cause of death among women worldwide (1). Most breast cancers are sporadic, whereas up to 10% are hereditary. Hereditary breast cancers tend to develop earlier in life than non-inherited (sporadic) cases and are more likely to develop in both breasts. In addition, multiple cancer diagnoses in consecutive generations and/or within the same subject, and transmission of gene alterations increase the risk of certain types of cancers to offspring (2). The most common cause of hereditary breast cancer is an inherited germline pathogenic variants (PVs) in the high-penetrant cancer predisposition genes *BRCA1* and *BRCA2* (3–5). A recent study on a large prospective cohort showed that the cumulative breast cancer risk to age 80 years was 72% for *BRCA1* and 69% for *BRCA2* carriers, and the cumulative risk for contralateral breast cancer 20 years after breast cancer diagnosis was 40% for *BRCA1* and 26% for *BRCA2* carriers (5). Advances in DNA sequencing techniques have helped to identify additional breast cancer susceptibility genes (4, 6). Among these genes, germline PVs in the Partner and localizer of BRCA2 (*PALB2*) gene appear to confer the highest risks (7). *PALB2* is located on chromosome 16p12.2, and encodes a protein which is essential for repair of double-strand DNA breaks by DNA homologous recombination (HR). The PALB2 protein acts as a linking hub of a macromolecular complex including *BRCA1* and BRCA2 and facilitates the function of RAD51, a protein vital for strand invasion during HR. PALB2 also interacts directly with and stabilizes BRCA2 during formation of the RAD51 nucleoprotein filament (8–11). Notably, PALB2 is essential to enable BRCA2 to perform its repair functions, which occur via HR, DNA double-strand break repair and S-phase DNA damage checkpoint control (8, 11). Not only PALB2 protein interacts with BRCA2 thanks to the domain at the C-terminus of PALB2, but it also binds to BRCA1 with its coiled-coil motif at the N-terminus. The disruption of this complex may result in efficiency decline of HR repair (12). In addition to promoting HR, PALB2 reduces the oxidative stress through its interaction with KEAP1. KEAP1, under normal condition, binds and inhibits the antioxidant transcription factor NRF2 leading to its degradation. PALB2 interacts competitively with KEAP1 causing NRF2 accumulation, thus regulating cellular redox homeostasis (13). Biallelic mutations in PALB2 result in a subtype of Fanconi anemia, which is a rare autosomal recessive syndrome characterized by genome instability, early bone marrow failure, growth abnormalities and increased cancer susceptibility. Otherwise, when PALB2 mutations occur as monoallelic, they are associated with predisposition to breast and other cancers (11).

*TP53, PTEN, SKT11, CDH1* and *NF1* are syndromic genes causing also relevant/high-risk for breast cancer. Inherited PVs in *CHEK2, ATM, BARD1, BRIP1*, and *RAD51D* were associated with moderate risks of breast cancer (6, 14, 15). Recently, *FANCM*, which encodes for a DNA translocase, has been suggested as a novel breast cancer predisposition gene, with greater effects for the ER-negative and triple-negative breast cancer subtypes (16, 17).

At clinical level, when germline PVs are identified in breast cancer susceptibility genes, primary or secondary prevention programs can be implemented, and tailored treatments initiated. Primary breast cancer prevention measures should be implemented through an accurate risk assessment. Primary prevention strategies are represented by chemoprevention with the selective estrogen receptor modulators (tamoxifen and raloxifen) or aromatase inhibitors (anastrozole and exemestane) (18) and by bilateral risk-reducing mastectomy (19). Secondary breast cancer prevention consists in screening programs based on clinical breast examination, mammogram, and contrast enhanced magnetic resonance imaging, aiming to detect pre-cancerous lesions and initial stage tumors. Here, we report a novel large germline deletion in *PALB2* in a young breast cancer patient who had a family history for different cancer types.

## Case Presentation

**Figure 1:**
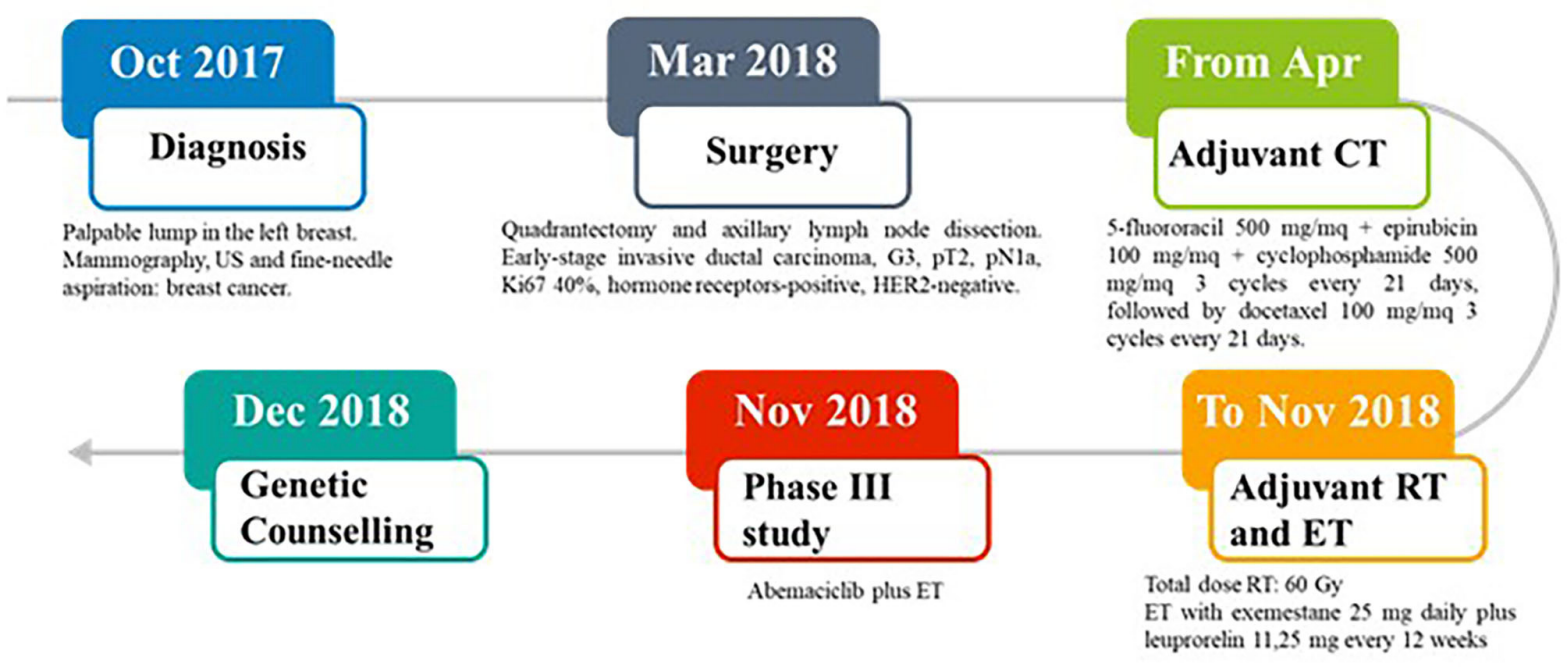
Case report timeline.

In October 2017, a 37-year-old Caucasian pre-menopausal woman without any relevant medical history felt a palpable lump in her left breast. Mammography combined with breast ultrasonography followed by fine-needle aspiration led to a diagnosis of breast cancer. In March 2018, she underwent a quadrantectomy and axillary lymph node dissection. Pathology exmination revealed a 31 mm, high-grade, luminal B-like invasive ductal carcinoma (estrogen receptor-positive, progesteron receptor-positive, HER2-negative, and Ki-67 40%). Two of 3 axillary lymph nodes were found to be metastatic. Bone scan, chest X-ray, and liver ultrasound examination did not identify distant metastases. In April 2018, the patient was started on adjuvant chemotherapy with 5-fluororacil 500 mg/mq + epirubicin 100 mg/mq + cyclophosphamide 500 mg/mq for 3 cycles every 21 days, followed by docetaxel 100 mg/mq for 3 cycles every 21 days. The patient then received adjuvant radiation therapy on the residual mammary parenchyma (total dose: 60 Gy) and adjuvant endocrine therapy (ET) was started with exemestane 25 mg daily plus leuprorelin 11,25 mg every 12 weeks. In November 2018, the patient was enrolled in an open-label, randomized, phase III study (NCT03155997) of abemaciclib plus standard ET (Figure 1).

**Figure 2:**
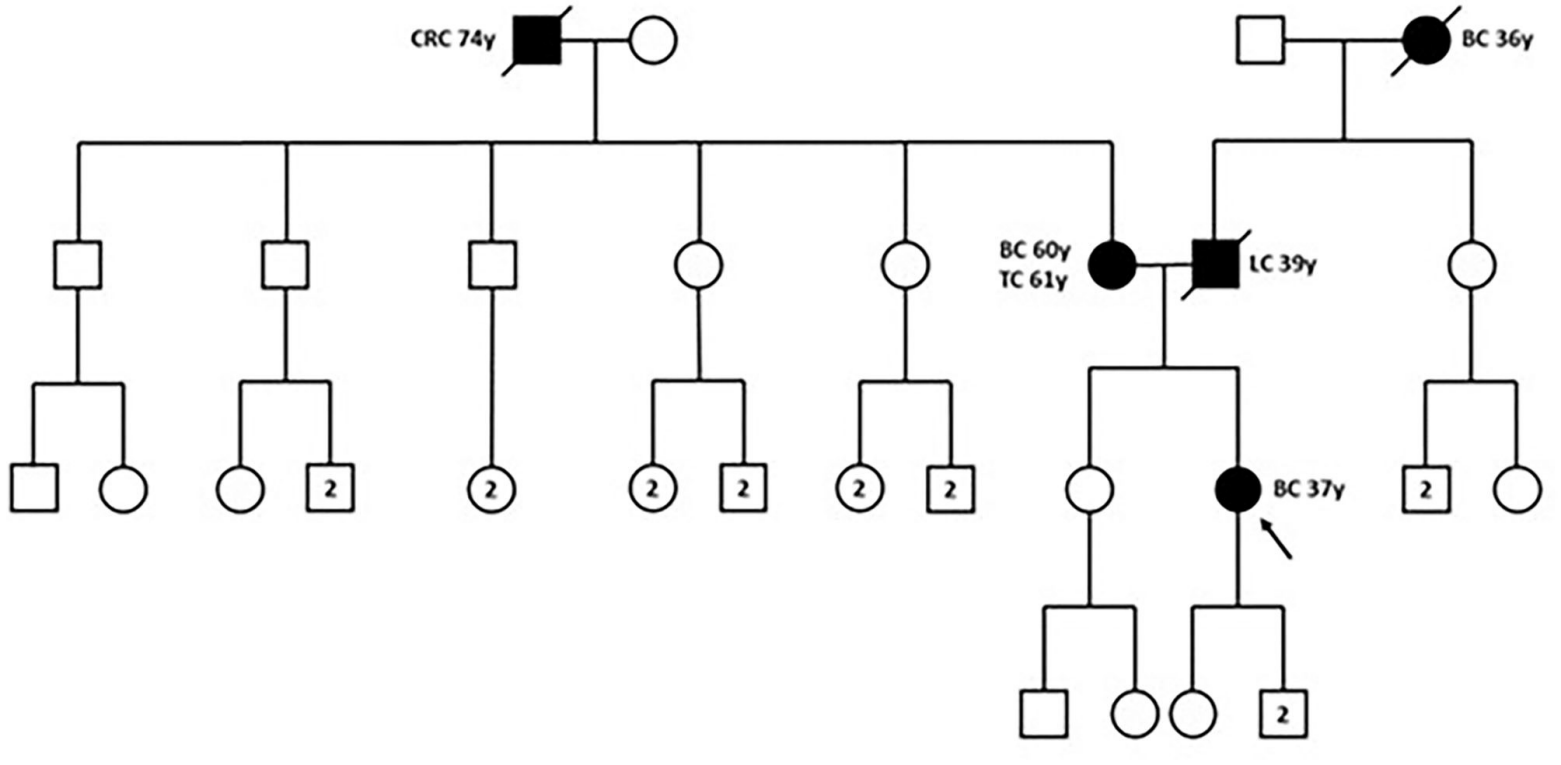
Family pedigree. The proband is indicated by black arrow. BC, breast cancer; CRC, colorectal cancer; LC, lung cancer; TC, thyroid cancer; y, years-old. Circle, female; square, male; filled symbols, individuals with cancer diagnosis; cross-hatched symbols, affected individuals already deceased; numbers in the circles/squares, amount of people of that gender.

**Figure 3:**
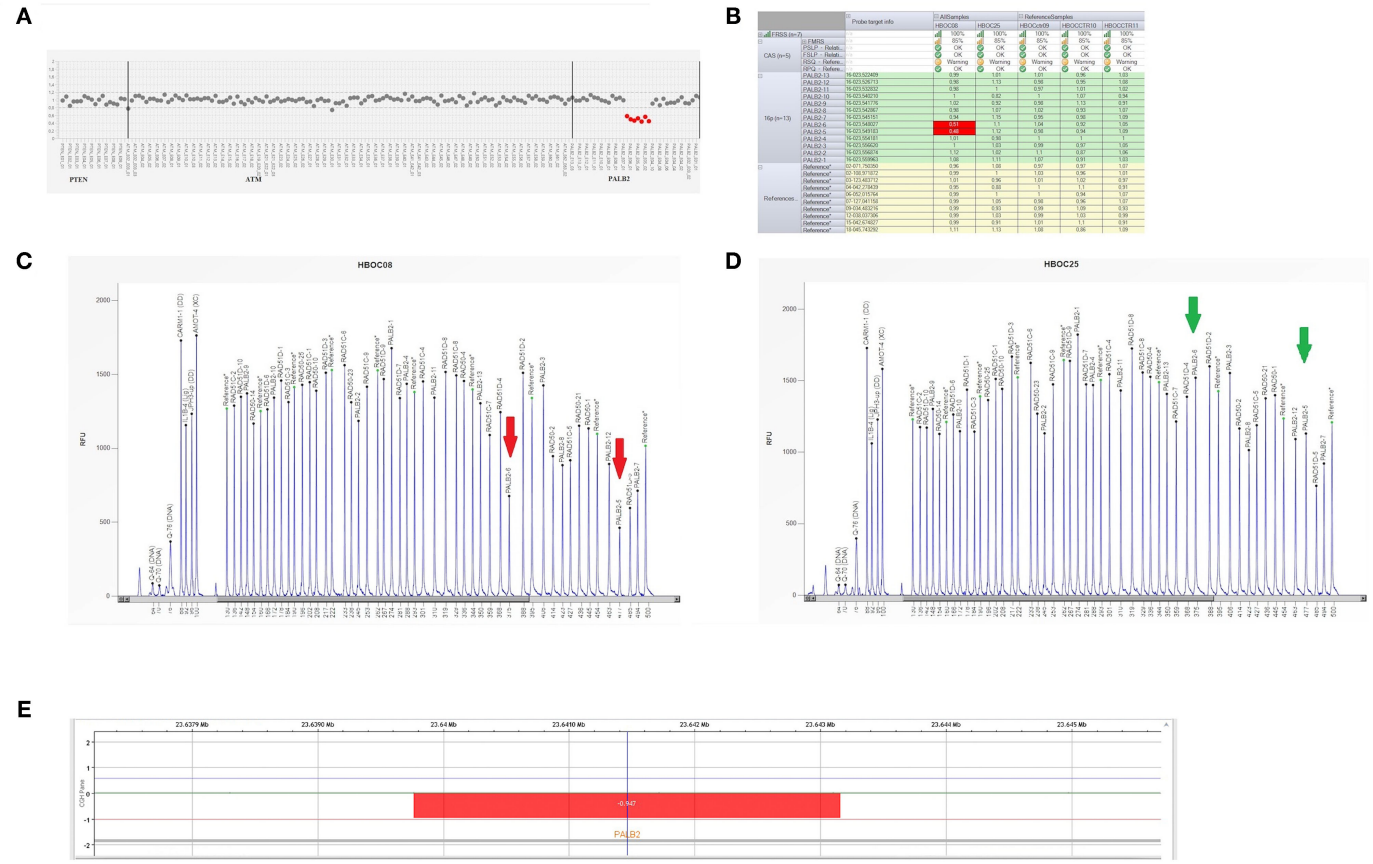
Genetic analysis of a *PALB2* gene. **(A)** Plot of *PALB2* amplicons corresponding to exon 5 and 6 which fell to about of 0.5 value of coverage. CNV analysis by Amplicon Suite Software for woman carrying the deletion of exon 5 and 6 of *PALB2*. Amplicon coverage: the coverage of the remaining *PALB2* exons was within the normal range (0.8–1.2), with a homogeneous pattern of distribution of each amplicon. **(B)** Drop of probe signals (red cells) of peak height area related to *PALB2* exons 5 and 6. **(C,D)** MLPA electropherogram from patient carrying the deletion of *PALB2* exon 5 and 6. Red arrows **(C)** indicate the deletions exon 5 and exon 6, respectively, as compared to the normal samples (green arrows) **(D)**. **(E)** CGH array profile of chromosome 16 from the Agilent 1X1M high resolution array revealed a heterozygous deletion in 16p12.2 region, of ~3.39 kb, involving *PALB2* gene.

In December 2018, the patient was referred to the Hereditary Cancer Genetics Clinic at the Department of Clincial Medicine and Surgery of the University Federico II in Naples, Italy, where she underwent genetic counseling. Family history revealed that her mother was diagnosed with breast cancer at the age of 60 and papillary thyroid carcinoma at the age of 61, that her paternal grandmother died at the age of 36 from breast cancer, whereas her father sucummbed to lung cancer at the age of 39, and her maternal grandfather died of colorectal cancer at the age of 74 (Figure 2). Genetic risk was assessed based on the medical and surgical history of the proband, the number of affected relatives, their age at dignosis and the degree of relationships among them. Since germline testing criteria were met (20), we looked for *BRCA1* and *BRCA2* gene PVs. Next-generation sequencing did not detect any pathogenic or likely PVs. Similarly, no deletions or duplications in exons of *BRCA1* and *BRCA2* genes were identified by multiplex ligation-dependent probe amplification (MLPA). Given the patient's personal and family history suggestive of inherited susceptibility, the patient underwent multi-gene testing with the Devyser HBOC NGS kit (Devyser, Hägersten, Sweden). The sequencing reaction was carried out on the Illumina MiSeq System (Illumina, San Diego, CA, USA). Output data were analyzed using the CE- IVD Amplicom Suite Software v. 1.0 (SmartSeq, Novara, Italy). As shown in Figure 3A, the software predicted the presence of a deletion in exons 5 and 6 of PALB2 gene. To verify the presence of this deletion, multiplex ligation-dependent probe amplification (MLPA) was performed (Figure 3B) by means of SALSA MLPA Probemix P260 *PALB2-RAD50-RAD51C-RAD51D* according to the manufacturer's instructions. In detail, four normal samples, previously screened by NGS in duplicate, served as reference samples for the MLPA test. Five μL of the proband's DNA were run in duplicate.

As shown in Figures 3C,D, the Q-Fragment plot of our sample confirmed the presence of a deletion in exons 5 and 6. To determine the extent of this deletion, we looked for large scale rearrangements using a high-resolution comparative genomic hybridization-array (a-CGH) (21). Genomic DNA was analyzed with the CGH 1 × 1M Microarray (Agilent Technologies, Santa Clara, CA, USA). The following [GRCh37] chr16p12.2 (23,639,765-23,643,159)x1 was investigated: results showed a 3.39 Kb deletion including the *PALB2* gene (chr16p12.2: 23,614,483-23,652,678), as obtained using the probes A_16_P20429174, A_16_P40587478, A_16_P20429183. As shown in Figure 3E, the deletion probably involved a 3.39 Kb region included between intron 4 and 6 causing the loss of exons 5 and 6.

To characterize the breakpoint region, specific PCR primers (Del4F 5′- aagactccactgactatctc-3′ and Del7R 5′-catcctgatgaaccactcatg-3′), including a larger region respect to that identified by probes used by CGH and amplifying a PCR product of 6.692 bp from the wild-type DNA, were designed. PCR reactions were performed using a long-range PCR kit (Expand Long Template PCR System, Roche Applied Science). Sequencing was performed using a BigDye Terminator Cycle Sequencing Kit v3.1 (Thermo Fisher Scientific) and an ABI 3500 Genetic Analyser (Thermo Fisher Scientific). Results were analyzed with the SeqScape v2.5 software package (Thermo Fisher Scientific) using NG_007406.1 reference. Total RNA was isolated from peripheral blood lymphocytes with TRIzol reagent (Thermo Fisher Scientific, Inc., Waltham, MA, USA). Synthesis of complementary DNA (cDNA) was performed with SuperScript II Reverse Transcriptase (Thermo Fisher Scientific) using DNAase-treated RNA in the presence of random primers and RNAaseOUT (Thermo Fisher Scientific). cDNA amplification was performed using the following primers: R4delF 5′-aggaagaagtcacctcacac-3′, and R7delR 5′-catcttcgcaagcagttatg-3′. Sequencing was performed using a BigDye Terminator Cycle Sequencing Kit v3.1 (Thermo Fisher Scientific) and an ABI 3500 Genetic Analyser (Thermo Fisher Scientific). Results were analyzed with the SeqScape v2.5 software package (Thermo Fisher Scientific) using NM_024675.3 reference.

**Figure 4:**
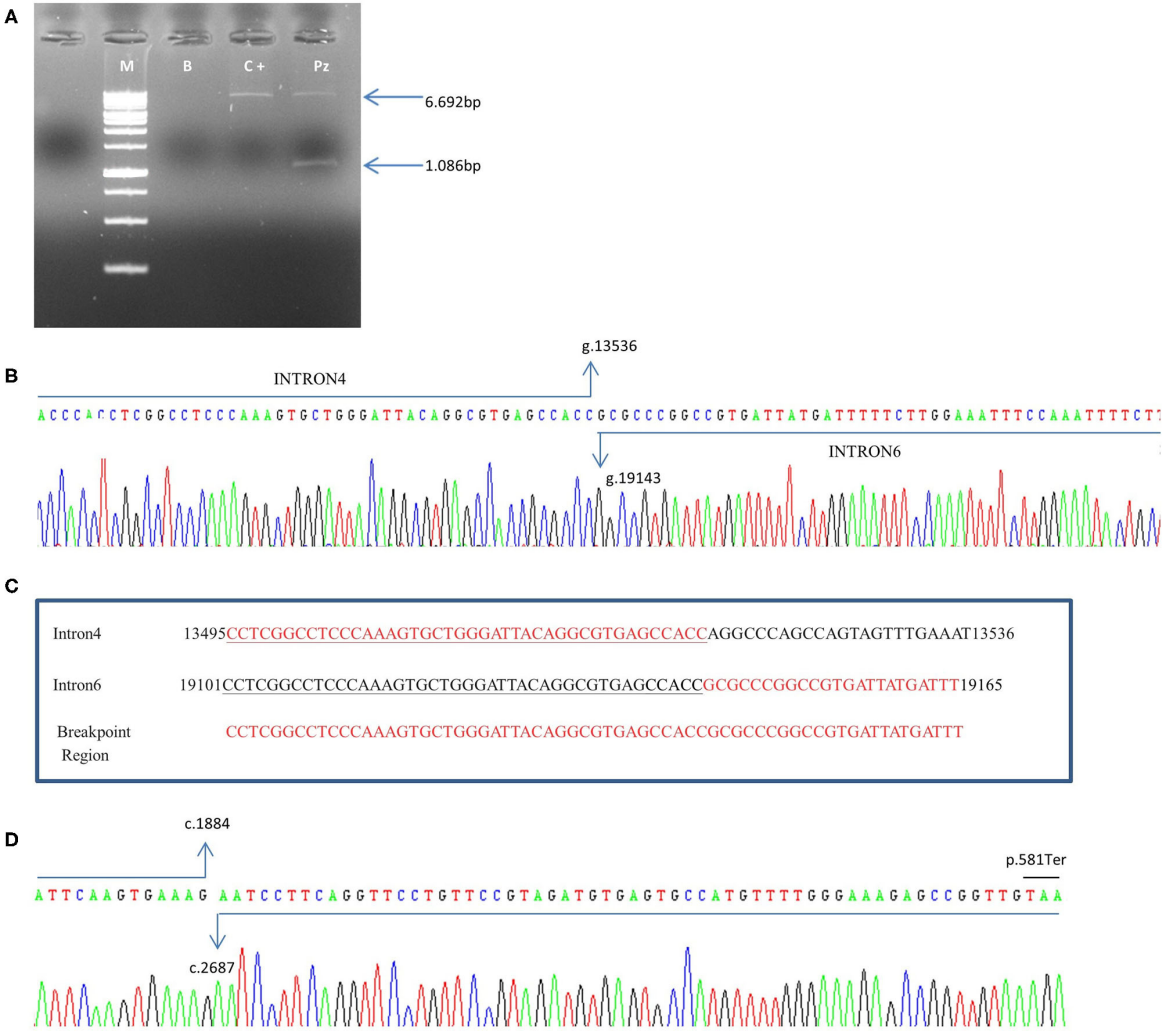
Characterization of exon 5–6 deletion in the *PALB2* gene. **(A)** Genomic DNA was amplified using specific deletion primers (Del4F and Del7R). The mutant allele gives rise to a specific 1.086-bp fragment. M: marker, B: blank, C+: wild-type control, Pz: patient. **(B)** The electropherogram of the 1.086 bp PCR fragment, containing the deletion breakpoint, showed a wild-type sequence until the nucleotide g.13536C (NG_007406.1) of *PALB2* intron 4. The following sequence corresponded to the *PALB2* intron 6 starting from the g.19143G nucleotide (NG_007406.1). **(C)** An homologous sequence of 43 nucleotides (underlined nucleotides), identified at breakpoint region, represents the cause of the rearrangement. **(D)** Electropherogram showing the sequence of the PCR product of 152 bp obtained from cDNA patient. Sequencing analysis revealed a wild-type sequence until the nucleotide c.1884G (NM_024675.3) of *PALB2* gene in exon 4. The following sequence corresponded to the *PALB2* exon 7 starting from the nucleotide c.2687A (NM_024675.3). A premature stop codon produces a truncated protein of 581 amino acids.

In the patient, two PCR products of 6.692bp and 1.086 bp respectively, were obtained (Figure 4A). The 1.086 bp fragment, containing the expected deletion, was cut out and isolated from agarose gel and sequenced using PCR primers Del4F and Del7R. This fragment, containing the breakpoint region, showed a wild-type sequence until to the nucleotide g.13536C (NG_007406.1) of *PALB2* gene intron 4. The following sequence corresponded to the *PALB2* intron 6 starting from the g.19143G nucleotide (NG_007406.1) (Figure 4B). We report the novel *PALB2* rearrangement in agreement with the recommended HGVS nomenclature: NG_007406:g.13536_19143del. The new deletion involves 5.607 bp of the *PALB2* gene, a larger deletion than the expected one of 3.39kb identified by the CGH, and includes part of the intron 4, exon 5, intron 5, exon 6 and part of the intron 6. Two sequences of 43 nucleotides, occurring in the same orientation (100% homology) within intron 4 and 6, suggested that the deletion is probably the result of a homologous recombination event (Figure 4C). A single PCR fragment of 1.052 bp was obtained from the cDNA control, while two fragments of 1.052 and 152 bp were amplified using cDNA patient. PCR product of 152 bp, containing the expected deletion, was cut out and isolated from agarose gel, sequenced with Del4F and Del7R primers, and analyzed. Sequencing analysis revealed a wild-type sequence until to the nucleotide c.1884G (NM_024675.3) of *PALB2* gene in exon 4. The following sequence corresponded to the *PALB2* exon 7 starting from the nucleotide c.2687A (NM_024675.3:c.1884_2687). The PALB2 exons 5-6 deletion, involving 803bp, disrupts the reading frame of the mRNA producing a pre-mature stop codon and a truncated protein of 581 amino acids [NP_078951.2:p. (Gly562GlufsTer21)] (Figure 4D).

## Discussion

Over the past years, several studies have shown that germline loss-of-function variants in the *PALB2* gene may confer an increased lifetime risk of breast, pancreatic, ovarian and other cancers. Rahman and colleagues identified *PALB2* monoallelic truncating variants in ~1% of patients with hereditary breast cancer (9) [as confirmed by Fernandes et al. (22)], whose risk of developing breast cancer was 2.3-fold higher than the risk observed amid controls. Subsequently, population-based screenings of *PALB2* PVs revealed a 2- to 30-fold increase in the risk of breast cancer (23–27). The analysis conducted by Antoniou and colleagues revealed that the relative risk for individuals with deleterious *PALB2* PVs was 8–9 before the age of 40 and around 5 after the age of 60 years. Thus, the cumulative breast cancer risk for female carriers of *PALB2* variants is 14% by the age of 50 years and up to 35% in women above the age of 70. Furthermore, *PALB2* is the most frequently altered gene (1.2%) in non *BRCA1*/*2* mutated male breast cancer patients, accordingly the risk of MBC increases from 9.63 to 17.30-fold in presence of *PALB2* pathogenetic variants (28). A study conducted in Poland in 2015 (29) evaluated the clinical outcomes of 116 *PALB2* mutation carriers among 12,529 women with breast cancer, and found that *PALB2* variants increased the risk of death from breast cancer. Indeed, 10-year survival was 48% in *PALB2* mutation carriers with breast cancer vs. 74.7% in non-mutation carriers with breast cancer and 72% in *BRCA1* mutated patients. In addition, survival differed in relation to tumor size. Indeed, 10-year survival was 82.4% in case of tumors smaller than 2 centimeters vs. 32.4% in case of tumors ranging from 2 to 4.9 centimeters (29). Notably, individuals with *PALB2* PVs more frequently had a triple-negative status (30%) vs. a frequency ranging between 12 and 17% in unselected patients with breast cancer (30). Yang et al. (7) analyzed 524 families from 21 countries who carried pathogenetic *PALB2* variants. They identified an association between mutated *PALB2* and female breast cancer (RR = 7.18), male breast cancer (RR = 7.34), ovarian cancer (RR = 2.91), and pancreatic cancer (RR = 2.37). The association between *PALB2* germline alterations and risk of developing pancreatic cancer has been also described by other groups. In 2009, Jones and colleagues carried out exomic sequencing of *PALB2* gene in 96 familial pancreatic cancer (FPC) patients in USA, thus identifying 3 truncating PVs producing different stop codons (31). Three more *PALB2* germline PVs producing truncated protein were identified among 81 European FPC family, each of these three family had also history of breast cancer (none of the patients was carrier of *BRCA2* mutation) (32). Furthermore, in a Japanese study, two out of 54 patients with pancreatic ductal adenocarcinoma carried deleterious variants of *PALB2* gene (33). In 2019, Janssen and colleagues provided a comprehensive catalog of *PALB2* gene predicted pathogenic or likely PVs published. They included 984 described cancer cases distributed over 146 *PALB2* predicted PVs. They observed that 911 (92.5%) cases were described in breast cancer patients, 49 (5.0%) cases in ovarian cancer patients, and 24 (2.4%) cases in pancreatic cancer patients. They also found that exons 2, 3 and 1 of *PALB2* gene showed the highest mutation rates (6.7, 5.8, 5.2%, respectively) (18).

Being a carrier of *PALB2* PVs has therapeutic and surveillance implications. As recommended by guidelines for the management of hereditary breast cancer of the American Society of Clinical Oncology, the American Society for Radiation Oncology and the Society of Surgical Oncology (34), the decision whether to apply local therapy or to perform a contralateral risk-reducing mastectomy in patients with breast cancer should not be based only on the presence of an alteration in a moderate-penetrance breast cancer gene, such as *PALB2*. If indicated, breast-conserving therapy may be a treatment option, followed by high-risk breast screening of remaining tissue with annual mammography and breast magnetic resonance imaging. Mammography and breast MRI should be alternated at 6 month intervals (34). Given the lack of data regarding the risk of developing contralateral breast cancer from moderate-penetrance genes, additional factors such as age at diagnosis and family history should be taken into account in selected patients (34). This would provide the best therapeutic option such as nipple-sparing mastectomy, bilateral mastectomy or contralateral risk-reducing mastectomy in patient previously undergone unilateral mastectomy. Despite some studies reported an association between *PALB2* PVs and the diagnosis of ovarian cancer (35–37), to date evidence are not sufficient to support risk-reducing salpingo-oophorectomy (20, 38). Similarly, there are no solid data regarding the systemic treatment options in *PALB2* carriers with breast cancer. Although *PALB2* encodes a protein involved in DNA double-strand break repair carried out by BRCA2, tailored therapies for breast cancer patients carrying PVs in genes other than *BRCA1/2* have not yet been established. However, the ongoing Olaparib Expanded phase II trial evaluated the effect of the poly ADP-ribose polymerase (PARP) inhibitor olaparib monotherapy in metastatic breast cancer patients with germline (other than *BRCA1/2*) or somatic PVs in DNA damage response (DDR)-pathway genes. In this trial, response to olaparib was observed mostly in patients with somatic *BRCA1/2* or germline *PALB2* PVs but not with *ATM* or *CHEK2* PVs. Among patients with *gPALB2* PVs, the overall response rate was 82% (90% CI, 53% to 96%), the clinical benefit rate was 100% (90% CI, 74–100%), and median progression-free survival (PFS) was 13.3 months (90% CI, 12 months to NA) (39). Other clinical trials are evaluating the potential role of PARP inhibitors in the treatment of *PALB2* PVs carriers with breast cancer (13, 41).

To the best of our knowledge, this is the first time that *PALB2* deletion involving a 5.6 Kb region, between intron 4 and 6 and causing the loss of exons 5 and 6, is reported as associated to a hereditary breast cancer. In addition, we also established the exact breakpoints of this new arrangement and estimated that introduces a pre-mature stop codon in *PALB2* mRNA resulting in the production of a truncated protein of 581 amino acids.

## Conclusions

Here we report a hitherto unknown *PALB2* 5.6-kilobase deletion involving exons 5 and 6 and the neighboring introns in a breast cancer patient. This case is paradigmatic of the clinical relevance of an in-depth evaluation of genetic risk, especially in patients with history highly suggestive of a hereditary syndrome. Consequently, patients without *BRCA1/2* alterations should be offered NGS multi-gene panel testing when personal and/or family history is suggestive for hereditary syndrome (40).

## Data Availability Statement

The original contributions presented in the study are included in the article/supplementary material, further inquiries can be directed to the corresponding authors.

## Ethics Statement

The studies involving human participants were reviewed and approved by Ethics Committee of the University of Naples Federico II. The patients/participants provided their written informed consent to participate in this study. Written informed consent was obtained from the individual(s) for the publication of any potentially identifiable images or data included in this article.

## Author Contributions

All authors of this research paper have directly participated in the planning, execution, or analysis of the study. All authors were involved in manuscript writing and reviewing, gave their final approval and agreed to be accountable for all aspects of the work.

## Conflict of Interest

CDA was a consultant/advisory board member for Novartis, Eli Lilly, and Pfizer. SDP had declared honoraria from Roche, Pfizer, Astra-Zeneca, Novartis, Celgene, Eli Lilly, Amgen and Eisai. The remaining authors declare that the research was conducted in the absence of any commercial or financial relationships that could be construed as a potential conflict of interest.
